# Supplementary material for: Association between polygenic risk and survival in breast cancer patients
Source: BMC Cancer. 2025 Aug 28;25:1393. doi: 10.1186/s12885-025-14640-9 (PMC12392544; doi:10.1186/s12885-025-14640-9)
Supplement: Supplementary file 1 — Supplementary Material 1. [file 12885_2025_14640_MOESM1_ESM.docx]

**Supplementary Figure 1. Kaplan-Meier survival curve for PRS_ER+**

**
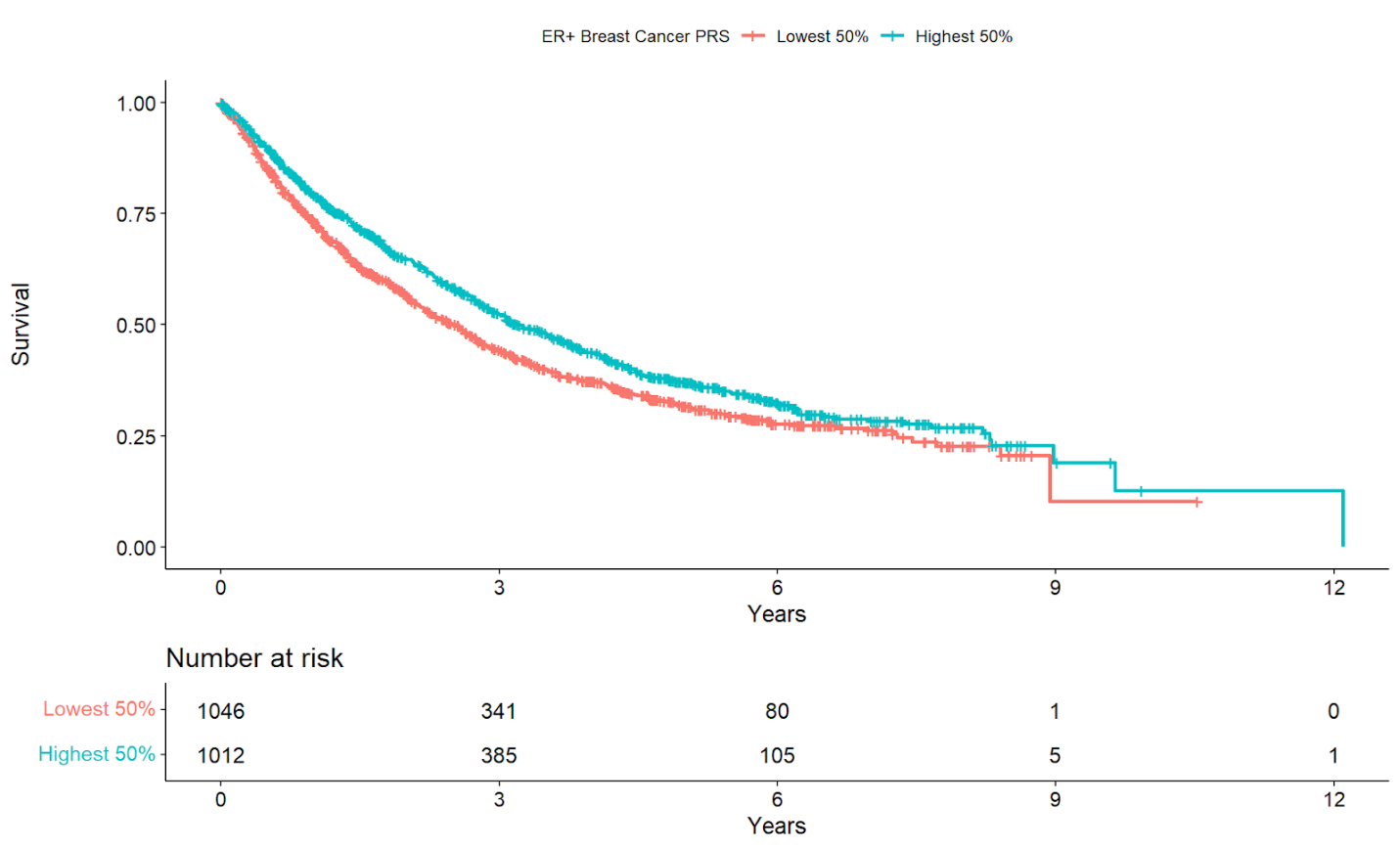
**Kaplan-Meier plot comparing survival of patients with PRS_ER+ scores in the highest 50% versus the lowest 50% of the cohort using the sequencing index date. Note that in the survival analysis, PRS was included as a continuous covariate.

**Supplementary Figure 2. Simulated ascertainment bias**


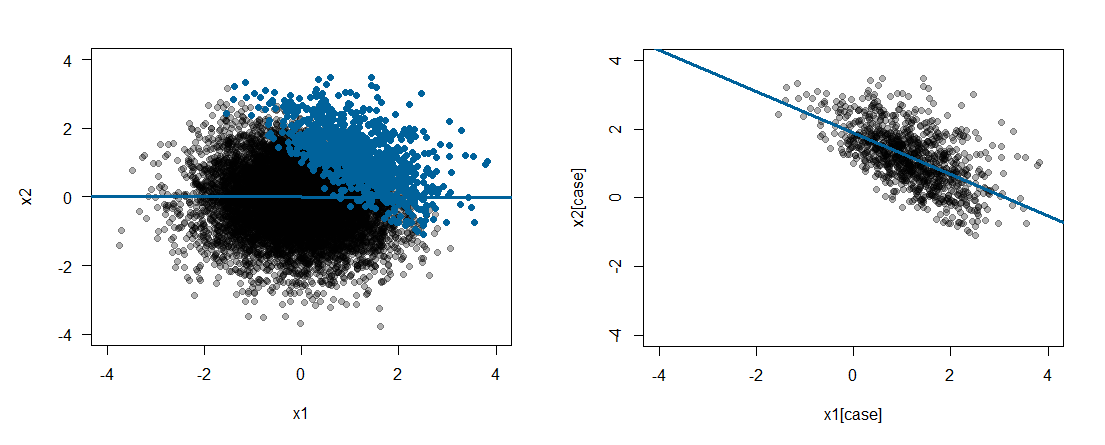
An example distribution of patients is shown on the left, with the top decile highlighted in blue. When we plot a regression on the entire population, no association is seen. However, when we limit our analysis to the top decile of patients, we see an association.

**Supplementary Figure 3. Relationships between genetic risk and exposure on cancer progression**


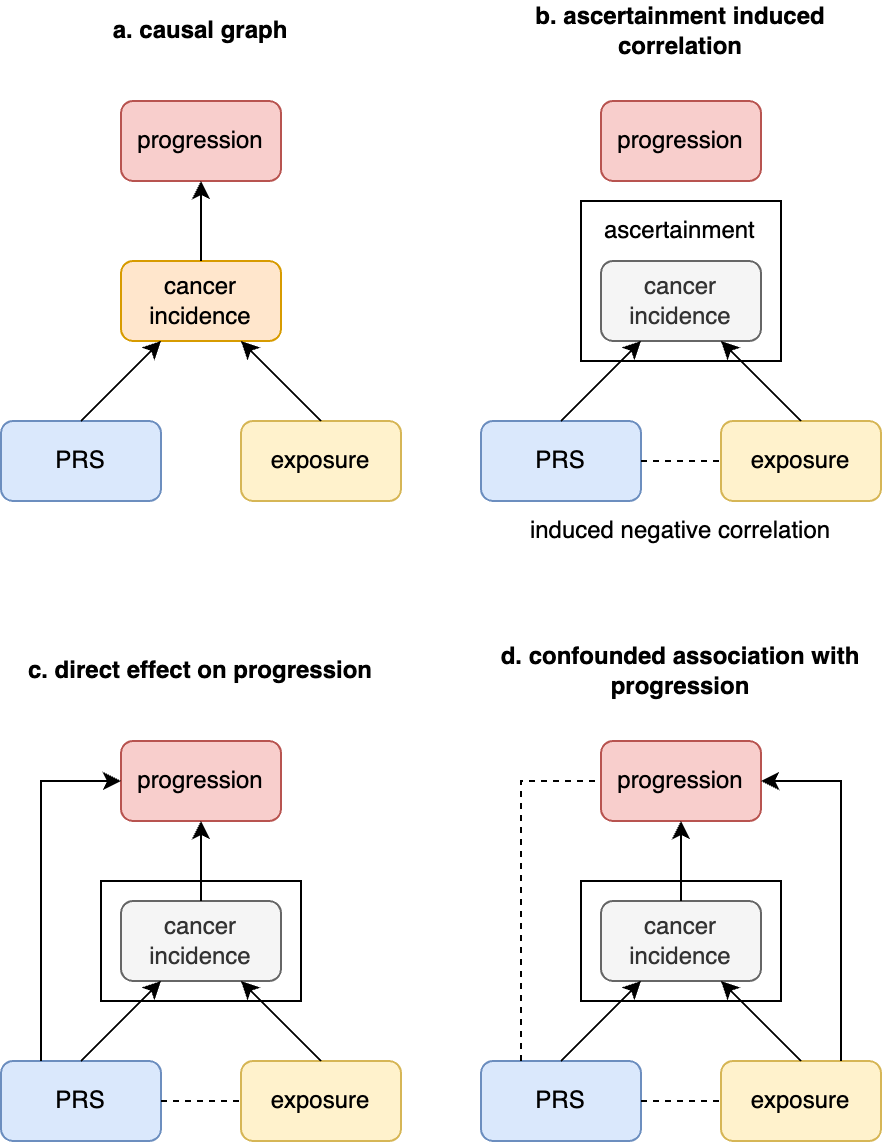


a) Both genetic risk (PRS) and environmental exposures contribute to cancer incidence. b) When we ascertain a cohort based on cancer incidence, we may identify an induced negative correlation between genetic risk and environmental exposure. c) Genetic risk may contribute to cancer progression independently of environmental exposure. This may appear to show a negative correlation between genetic risk and environmental exposure. d) Where environmental exposure contributes independently to progression, we may see an induced negative correlation between genetic risk and progression, and genetic risk and exposure.

**Supplementary Figure 4. Comparing raw and bias-adjusted SNP effects**


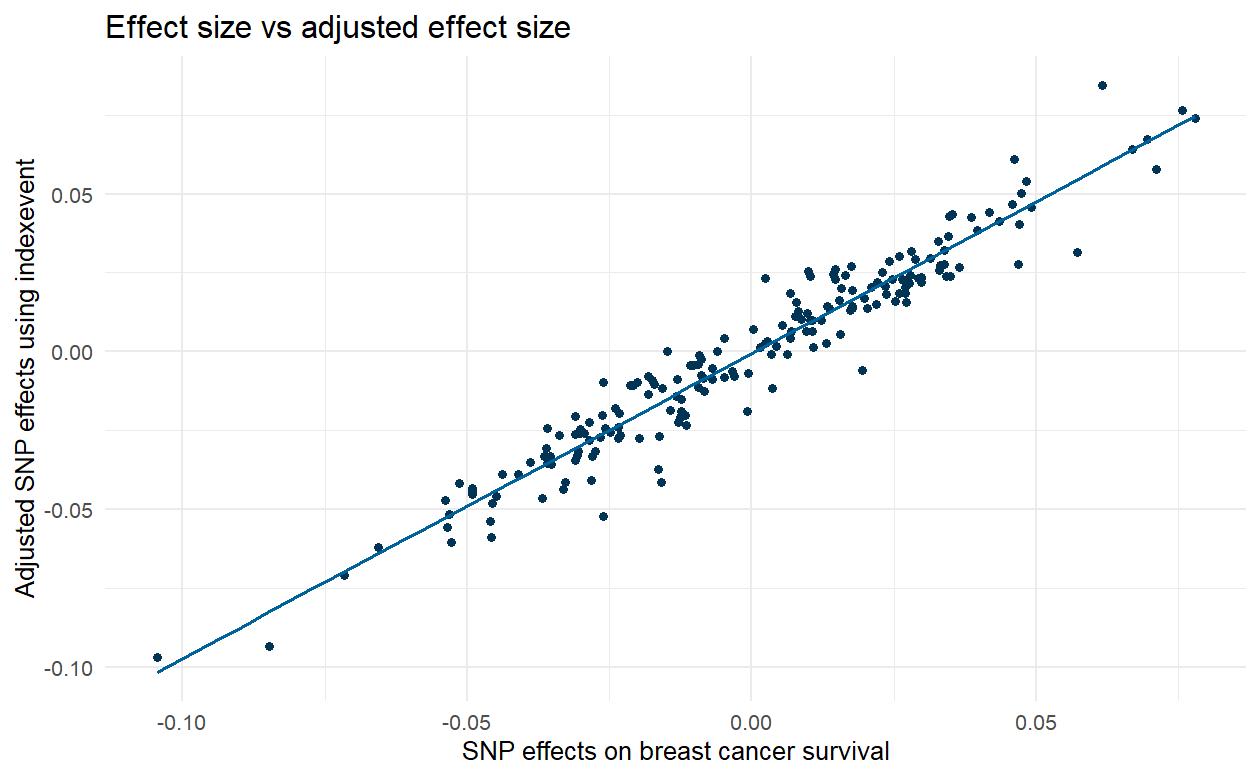


Comparison between raw vs corrected SNP effects on breast cancer survival using the indexevent package in R.
